# Development of live cultural pandemic influenza vaccine Vector-Flu

**DOI:** 10.1186/1753-6561-5-S8-P104

**Published:** 2011-11-22

**Authors:** Elena A Nechaeva, Tatyana Y Sen’kina, Alexander B Ryzhikov, Irina F Radaeva, Ol’ga G P’yankova, Natal’ya V Danil’chenko, Tatyana M Sviridenko, Marina P Bogryantzeva, Natal’ya V Gilina, Nikolay A Varaksin, Tatyana G Ryabicheva, Irina V Kiseleva, Larisa G Rudenko

**Affiliations:** 1 Department of Cell Culture Technology, SRC VB VECTOR, Novosibirsk, 630559, Russia; 2 SRC VB VECTOR, Novosibirsk, 630559, Russia; 3 Institute of Experimental Medicine, Russian Academy of Medical Sciences, St. Petersburg, 197376, Russia

## Background

The threat of pandemic A/H1N1 influenza remains a matter of considerable public concern. Recent influenza outbreaks underline the importance of rapid production of a reserve of vaccine sufficient for pandemic and interpandemic periods. Traditional vaccines intended for seasonal influenza do not satisfy this demand, because they are unable to induce cross-reactive antibodies to pandemic flu.

Live influenza vaccines made in the cell culture offer potential advantages because it is possible to produce a considerable amount of these vaccines over a short period. Their use eliminate concerns about allergic reactions to egg proteins, and they allow the antigenic composition of the vaccine to be closer to the circulating influenza virus strains.

The goal of this work was to develop a live attenuated vaccine against pandemic influenza, Vector-Flu, derived from cold-adapted strain A/17/California/2009/38(H1N1) and to study the immunogenic and protective properties of the vaccine using ferrets and mice.

## Materials and methods

### Cell culture

MDCK from Cell Culture Collection of SRC VB VECTOR. Cells were passed in serum-free SFM4MegaVir medium (USA). The characteristics of the MDCK cell line were studied in accordance with WHO [1].

### Viruses

The vaccine strain A/17/California/2009/38(H1N1) was generated at the Institute of Experimental Medicine (St. Petersburg, Russia) by reassortment of the cold-adapted attenuated A/Leningrad/134/17/57 (H2N2) master donor virus with the pandemic strain A/California/7/2009 (H1N1). The A/Chita/3/2009(H1N1) influenza virus was obtained from VECTOR’s Collection of Microorganisms.

### Animals

Female ferrets were 4–5 month old and were obtained from the Animal Farm Rodniki (Pushkino, Moscow region, Russia).

### Determination of influenza virus infectious activity

The infectious activity of influenza virus was determined by titration in 10–12-day-old chick embryos. 10-fold dilutions (0.2 ml) of virus-containing fluid were inoculated into the allantoic cavity of chick embryos. The embryos were incubated for 48 hours at a temperature of 35°C. After the incubation, the allantoic fluid was harvested from the embryos to determine the virus infectious activity by agglutination reaction with 1% chicken red blood cells. The virus titer was calculated according to the Reed–Muench method and expressed as log EID_50_/0.2 ml [2].

### Control of immunogenicity of the Vector-Flu vaccine

18 ferrets were anesthetized to take blood samples (5 ml) from the caudal vein; they were then immunized with 200 µl of the Vector-Flu vaccine at 6.6–6.8 log EID_50_ dose delivered into each nostril. One ferret subgroup was immunized with a single dose and another subgroup received two doses of vaccine with a 10-day interval between vaccinations. At day 21 after the last immunization, the sedated animals were bled from the caudal vein to obtain sera and the antibody titers were determined by hemagglutination inhibition and microneutralization assays.

*Hemagglutination Inhibition* (*HAI*) *test.* HAI was performed by a routine technique [3] with some modifications. The assayed sera were pre-treated with the receptor destroying enzyme (RDE). The hemagglutination reaction was performed with 1% chicken red blood cells (RBC). The HAI titer was determined as the reciprocal dilution of the last row which contained non-agglutinated RBC.

*Microneutralization assay.* The assay was performed in compliance with the WHO guidelines [3] with some modifications. MDCK cells supplemented with equal volumes of serum and influenza virus were mixed and incubated in 5% CO_2_ at 37°C. The presence of the virus was detected by enzyme immunoassay using the monoclonal antibodies to type A influenza virus NP protein (CDC, Atlanta). Neutralizing antibody titer was defined as reciprocal of the highest serum dilution that provided 50% inhibition of the virus growth in cell culture.

## Results

The live MDCK-derived Vector-Flu vaccine was produced in accordance with the established production specifications. Ever in Russia, based on MDCK cell line, we have developed seed and working banks of cells; cell banks are certified in accordance with WHO recommendations [1].

The vaccine production technology involved the following steps:

• microcarrier cultivation of MDCK cells in fermenters in serum-free medium;

• A/17/California/2009/38 (H1N1) virus growth in the cell culture;

• virus-containing fluid collection and purification;

• introduction of stabilizers and biodegradable polymer;

• freeze-drying; ready-to-use vaccine formulation production.

7 laboratory batches of vaccine Vector-Flu have been produced and certified; they have specific activity index as 7.33-7.5 lg EID_50_/ml.

Ferret immune response data to administration of the live MDCK-derived Vector-Flu vaccine are listed in the Table 1. High serum titers of the ferrets immunized with A/H1N1 pandemic influenza virus strain (up to 1:5120 by HAI after a single dose vaccination) should be noted. Second dose vaccination with the A/17/California/2009/38 (H1N1) vaccine strain increased the HAI titers from 1:1097 to 1:1493, while the geometric mean titers (GMTs) determined by microneutralization assay, went from 1:1016 to 1:2032, respectively. The Vector-Flu vaccine is immunogenic against both A/Chita/3/2009 strain of pandemic A/H1N1 influenza virus isolated in Russia, and the pandemic influenza virus strain A/California/7/2009.

**Table 1:** The titers of ferret blood serum to A/H1N1 influenza virus strains before and after immunization with the Vector-Flu vaccine determined by HAI and microneutralization assay.

Serological method	HAI						Microneutralization					
Influenza virus strain	A/Chita/3/2009			A/California/7/2009			A/Chita/3/2009			A/California/7/2009		

Number of vaccinations	0	1	2	0	1	2	0	1	2	0	1	2

GMT^a^ over the group	<20	2987	2560	<20	818	3556	<40	1636	5120	<40	924	1422

SD of the GMT		1693	1568		718	1538		723	0		649	699

^a^Mean geometric titer

We have developed the vaccine production pilot schedule and have prepared the set of specification documents for vaccine registration application submission to Ministry of health and social development of Russia. We are planning to do the clinical trials Phase I in Russia in 2011.

## Conclusions

We have developed live cultural influenza vaccine production technology. The technology used for pandemic influenza vaccine Vector-Flu production has a versatile use mode, and it could be used for growing live cultural vaccines based on the other influenza virus subtypes. We have obtained the patent of Russia #2330885 for the developed technology.
